# Hospital Meetings, &c.

**Published:** 1900-08-11

**Authors:** 


### HOSPITAL MEETINGS, &C.

### HOSPITAL FOR EPILEPSY AND PARALYSIS.

Site Acquired and Half the Money Raised for the New Building.

The annual meeting of governors and supporters of the Hospital for Epilepsy and Paralysis and other Diseases of the Nervous System was held on Wednesday, July 25th, at the hospital, Portland Terrace, Regent's Park, W. The President (the Earl of Hardwicke) occupied the chair, supported by Bishop Barry, D.D.

The managing committee's annual report, which was read by the Secretary, stated with reference to the lease of the present building, which had been occupied by the hospital since 1872, that by an arrangement with the purchaser of the remainder of the lease the hospital would continue in occupation of the premises till October next. This extension had given the committee time to further consider their future plans; but they had not succeeded in obtaining a building in which the work of the hospital could be carried on, and the erection of a new building was therefore forced upon them. This did not for a moment appear regrettable, but the stress of the position lay in its extreme financial difficulty. At a time when the claims made by the South African War and the Indian Famine were engrossing the living it was most providential that the hospital should, during the last few years, have been indebted, in a degree quite without precedent, to the dead. In addition to the legacies of £1,000 and £900 received in 1897 and 1898 respectively, the bequests in 1899 reached £3,170, while recently £5,000 had been received from the executors of Mr. J. W. N. Bentley out of the residue of his estate bequeathed to them for distribution, and a grant of £100 similarly awarded by the Rev. J. F. Anstey under the will of the Rev. J. Morgan. These large and unexpected additions to the funds had led the committee to feel justified in the steps they had taken towards the acquisition of a site and the preparation of plans for a new hospital. The acquirement of the site for the building, and the cost of erection and furnishing it, would require at least £25,000, of which only £12,600 was in hand. The difficulty of obtaining a suitable site had been great, and had only recently been surmounted by the completion of the negotiations for a most suitable piece of land in an excellent and accessible position in the immediate neighbourhood of the present hospital. This, the committee trusted, would soon be in their possession, on the terms, however, of a long building lease, since it was found impossible to obtain a freehold site of suitable position and character. It would not, of course, be possible to utilise the site until the new building was ready, but the committee hoped that they might be able to provide for the work of the out-patient department by the acquisition of temporary premises. The necessary plans and drawings for a building to contain a minimum of twenty-five beds, a suitable out-patient department, and all other primary essentials were being prepared by Mr. Keith D. Young, F.R.I.B.A., whom the committee had selected from among twelve architects. Last year the committee received a grant of £250 from the Prince of Wales's Hospital Fund for the building fund on condition that the new hospital was commenced in 1900, a condition with which the committee had no doubt they would be able to comply. The award was peculiarly welcome as a proof that the council appreciated the work of the hospital and the provision which the committee were now making for its continuation and re-establishment on a proper basis. With such views, and in consequence of certain business arrangements in connection with the acquisition of a site, the committee had procured the incorporation of the hospital under the provisions of the Companies' Acts by licence of the Board of Trade. The president, treasurer, and two members of the committee of this hospital appended their signatures to the memorial presented to the Local Government Board praying for the introduction of a Bill to relieve hospitals from local rates. The committee received with regret the resignation of Dr. Althaus as a member of the committee, which position he had occupied since the establishment of the hospital in 1866. Dr. Althaus died on June 11th last, and by his death the hospital was deprived of its founder, who was also, until May, 1894, its senior acting physician, and, from that time to the end of his life, was on its consulting staff. The committee had thought it only right to treat £9,900 of the bequests already enumerated as capital, and to place that sum to the account of the new building fund for future investment, so to speak, in the permanent form of an erected building. The remainder of the legacies had been treated, as before, as an item of the general receipts, and had in part prevented the necessity for a further sale of stock. This was only, however, avoided by the loan of £100 by two members of committee, which had since been repaid. The sum of £116 18s. 3d. was ultimately paid under the will of Mrs. E. Armitage, who bequeathed to this and other hospitals £4,000 each, but the available residue was not large enough to permit of a nearer approach to her intentions in any such case. Donations to the general fund showed a slight increase, but the annual subscriptions exhibited a considerable diminution. Out of a total loss of £43, £30 was caused by the death of subscribers. Of £3,170 received under wills in 1899, £3,000 was a specific bequest from Miss S. Davis. Since January 1st, 1897, the total amount of legacies received amounted to £10,361, of which £4,186 was specifically bequeathed to the hospital, and £6,175 awarded by executors. The sums of £67 and £37 were received from the Hospital Sunday and Hospital Saturday Funds. The number of in-patients under treatment in 1899 was 105, or two less than last year, a number identical with the average of three preceding years, and the average length of residence was 65 days. There was one death, resulting from disseminated sclerosis. The attendances of out-patients during 1899, though not so numerous as in the preceding year, were considerably in excess of the average of the five years ended in 1896, being 11,259, compared with 9,245. The balance-sheet showed that the annual subscriptions were £330, donations £376, patients' payments £507, ordinary income amounting to £1,348, legacies (represented by £3,170 of the total receipts) were £4,856. The total ordinary expenditure (£1,634) was increased to £4,856 by extraordinary expenditure.

The Chairman, in moving the adoption of the report, said that as president of the meeting last year he had a lively recollection of the position they had to face on that date. The outlook was by no means rosy. Their resources were not great, and it appeared absolutely unavoidable for the hospital to change its premises at the beginning of this year. At that meeting they had to make up their minds whether the good work this hospital had done in the past should be discontinued, or whether they should use their best endeavours to acquire new premises to continue the work which had been carried on for so many years. All present were unanimously agreed that the work should be carried on, and resolved to do the best they could to secure the necessary funds to meet the difficulties which confronted them. As the report clearly showed, those difficulties had been met in a very satisfactory manner. He did not say it was altogether due to those connected with the hospital, because they had had a good deal of luck, but at the same time he thought that the committee and secretary were entitled to the most cordial congratulations because they had left no stone unturned to secure the necessary support, and their efforts had met with no small success. The matter of securing a suitable site was very difficult, and their secretary had personally visited all parts of London in the search. They had now £12,500 towards the amount required for the new building, which was exceedingly satisfactory. They had been fortunate to receive a donation from the Prince of Wales's Hospital Fund, which showed that the work of the hospital was appreciated, because he knew that great discrimination was exercised by the Council of the Fund in the distribution of its moneys. He thought that this recognition of the hospital should encourage them to further efforts to obtain the sum now required. He thought the incorporation of the hospital was a wise step, as it afforded certain facilities in the negotiations for acquiring the site. As to the rating of hospitals, he was very pleased to append his name to the memorial to the Local Government Board praying that such institutions should be relieved from paying rates. He believed that if only sufficient energy was shown by those interested in the matter hospitals would in the future be excused. (Hear, hear.) Referring to the death of Dr. Althaus, the Chairman said they all regretted the death of one who had at all times shown the greatest interest in the hospital, and whose distinguished services had so greatly contributed to the work in hand. (Applause.) He felt sure that Dr. Althaus would have supported the proposals made in the report and the efforts put forth to raise the money for erecting a hospital on such lines and of such dimensions as were necessary if they were to carry out the work which he inaugurated, and which he would have continued to follow with the deepest and keenest interest. The new hospital would greatly facilitate the work of the doctors. Almost every day fresh experiments and discoveries of medical science were taking place, and if the new hospital could only progress in the same way as the old one had done, and if they could continue to relieve suffering and to find new cures for the special illnesses of the patients admitted, he believed they would be carrying on a great and necessary work, and at once worthy of the best record of this hospital. (Applause.)

Bishop Barry seconded the motion, and spoke of the necessity of such an institution for the treatment of nervous diseases, which he was afraid were on the increase owing to the fact that we lead a faster life than previous generations. He believed that the institution had carried on its work most successfully for some years, and it was this success which had led to their difficulties, for success was sure to open out larger ideas and aspirations. Therefore he was not surprised to hear that their success had brought upon them the necessity of facing a great crisis. They had done a good deal in that direction. He understood that there was enough money subscribed to make a start with the building, but he could understand that the more money they had to start on the cheaper it would be in the end. Still their position was obviously sound and healthy; there were few hospitals like theirs out of debt. Of course this hospital was only part of the great hospital work of our great city. The hospitals performed what was nothing less than a national service in that it told on the wellbeing of society and maintained medical skill and science. It was a service of humanity, for it encouraged sympathy between various classes, and was also a great Christian work. For these three reasons he commended the hospital to their support.

The motion was carried unanimously, and a vote of thanks to the chairman concluded the proceedings.

